# Fabrication of a metal free catalyst for chemical reactions through decoration of chitosan with ionic liquid terminated dendritic moiety

**DOI:** 10.1038/s41598-020-76795-8

**Published:** 2020-11-12

**Authors:** Samahe Sadjadi, Fatemeh Koohestani, Majid M. Heravi

**Affiliations:** 1 Gas Conversion Department, Faculty of Petrochemicals, Iran Polymer and Petrochemical Institute, PO Box 14975-112, Tehran, Iran; 2 Department of Chemistry, School of Science, Alzahra University, Vanak, PO Box 1993891176, Tehran, Iran

**Keywords:** Chemistry, Catalysis

## Abstract

In attempt to develop a biocompatible metal-free catalyst, a dendritic moiety was grown on chitosan through successive reactions with 2,4,6-trichloro-1,3,5-triazine and ethylenediamine. Subsequently, the terminal functional groups of the dendron were decorated with 1-methylimidazolium chloride. The catalyst was characterized with SEM, EDS, TGA, FTIR, XRD and mapping analysis. Then, the catalytic activity of the resultant composite was scrutinized for catalyzing Knoevenagel condensation and synthesis of xanthene derivatives in aqueous media under mild reaction condition. The results confirmed high activity of the catalyst, superior to ionic liquid free counterpart and bare chitosan. This observation was ascribed to the instinct catalytic activity of ionic liquid. Moreover, using control catalysts, it was confirmed that the presence of the dendritic moiety that could increase the content of ionic liquid on the backbone of the catalyst enhanced the catalytic activity.

## Introduction

The popularity of carbohydrates for the catalysis lies in the availability, bio-compatibility, biodegradability, tune-ability and diversity of these compounds. In fact, both linear and cyclic carbohydrates have been successfully utilized for catalytic purposes^1–3^. In this regard, carbohydrates can be applied as catalysts or potential supporting materials for the stabilization of other species^4–6^. One of the carbohydrates that can be found in large quantity is chitosan, CS. This carbohydrate that can be furnished by deacetylation of chitin, benefits from the presence of amino group in its structure. As this functionality can be readily reacted with other reagents to provide more complex functionalities, CS has been extensively applied for devising catalytic composites^7,8^. Similar to other supporting materials, introduction of ligands or other functional groups^9–11^ can improve the performance of the support and provides opportunities for incorporation of catalytic active sites^12^.

Ionic liquids, ILs, are organic salts with organic cations and organic or inorganic anions^13–15^ that possess low melting points and vapor pressures^16–18^. ILs can be prepared through conventional chemical reactions and the diversity of the possible cations and anions provides an opportunity to design customized IL via combinations of different components^16,19^. Excellent properties of ILs, such as their tunable polarities, low toxicity, high thermal stability, non-flammability, and adjustable solvation expanded their uses in various domains, especially catalysis^17,20,21^. To date, various chemical transformations have been catalyzed by these environmentally benign catalysts. The main goal of our research group is developing heterogeneous catalysts by using natural compounds such as carbohydrates and natural clays^22–27^. Considering the environmental concerns and economic issues, use of metal-free catalysts received great attention. In this regard, use of ILs can be a promising solution. However, the homogeneous nature of IL renders its recovery and recyclability tedious. To circumvent this issue, supporting IL on a catalyst support is suggested. In this context, use of CS as a natural and biocompatible compound is very appealing.

**Figure 1:**
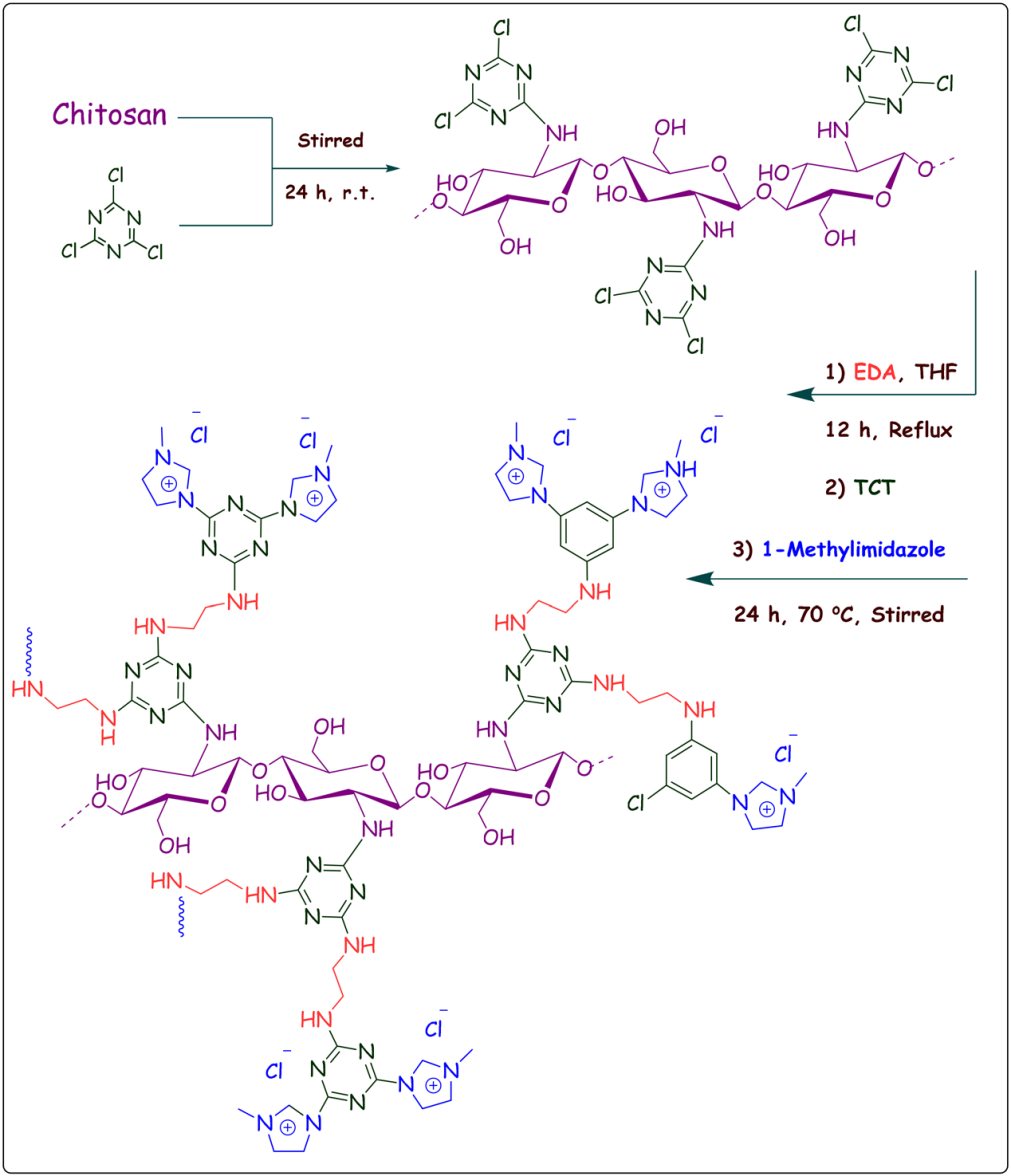
Synthetic procedure of CS-D-IL.

We are interested in using carbohydrates for designing heterogeneous catalysts^22–27^. Our studies confirmed the possibility of introduction of dendritic moiety on supporting materials such as clays for improving the performance of the support^9^. On the other hand, our recent work indicated that by introduction of a dendritic moiety on bentonite clay, loading of IL can be improved^12^. Those promising results encouraged us to investigate whether conjugation of a dendritic moiety on CS can also be helpful for designing metal free catalysts with appropriate IL content. Hence, in this article we report a new catalytic carbohydrate-based composite that benefits from the chemistry of CS, dendron and IL. In fact, it is assumed that IL can act as the main catalytic active site and dendrimer decorated CS can serve as a support for heterogenation of IL and improving its loading. To fabricate the composite, CS was first functionalized with 2,4,6-trichloro-1,3,5-triazine and then successively reacted with ethylenediamine and 2,4,6-trichloro-1,3,5-triazine to graft a dendron on CS. Finally, the terminal Cl groups of the dendron were reacted with 1-methylimidazole to decorate the dendron with ILs, Fig. 1. The resultant composite was then utilized as a bio-based and metal free catalyst for catalyzing Knoevenagel condensation of aldehydes and malonitrile in aqueous media at ambient temperature and synthesis of xanthene derivatives. The reaction condition was optimized and generality of this protocol was investigated by applying various aldehydes with different electronic features. Furthermore, the roles of IL, dendritic moiety and CS in the catalysis was studied. At the end of this research, the recyclability of the catalyst was appraised.

## Result and discussion

### Verification of formation of CS-D-IL

**Figure 2:**
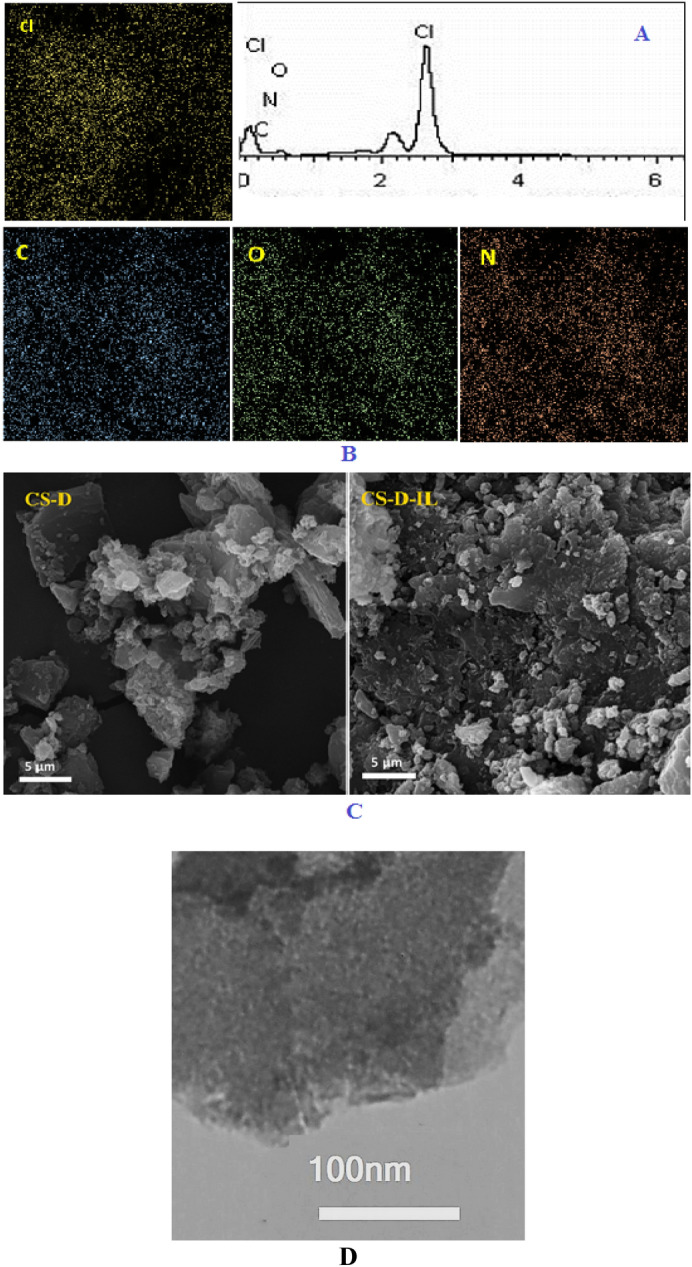
(**A**) EDS analysis, (**B**) elemental mapping analysis, (**C**) SEM images of CS-D and CS-D-IL and (**D**) TEM image of the catalyst.

The first performed analysis to establish the incorporation of TCT, EDA and IL, was EDS analysis. First, CS and CS-D were analyzed via EDS, Fig. S1. In EDS analysis of CS, only C, O and N atoms were present. These atoms are representative of CS. In the case of CS-D, apart from C, O and N atoms, Cl atom was also observed. This issue can confirm conjugation of TCT. Similarly, in the EDS analysis of CS-D-IL (Fig. 2A), C, O, N and Cl atoms are detected. In fact, Cl atom not only can be ascribed to the counter ion of IL, but also can be indicative of some Cl functionalities of TCT structure that did not have a chance to react with 1-methyl imidazole due to the steric hindrance. Moreover, C and N atoms can also approve the incorporation of EDA, TCT and IL. Next, the elemental mapping analysis was carried out, Fig. 2B. As depicted, all atoms showed high dispersion. Therefore, it can be concluded that IL terminated D was uniformly grafted on CS. The morphology of CS-D and CS-D-IL were investigated via SEM analysis, Fig. 2C. As the comparison of the two SEM images shows, CS-D and CS-D-IL exhibited distinguished morphologies. In fact, CS-D-IL showed more packed and aggregated morphology compared to CS-D. This issue can be due to the non-covalent interactions of ILs that trigger formation of aggregates. The morphology of CS-D-IL was also investigated via TEM, Fig. 2D. As expected, CS-D-IL was amorphous.

**Figure 3:**
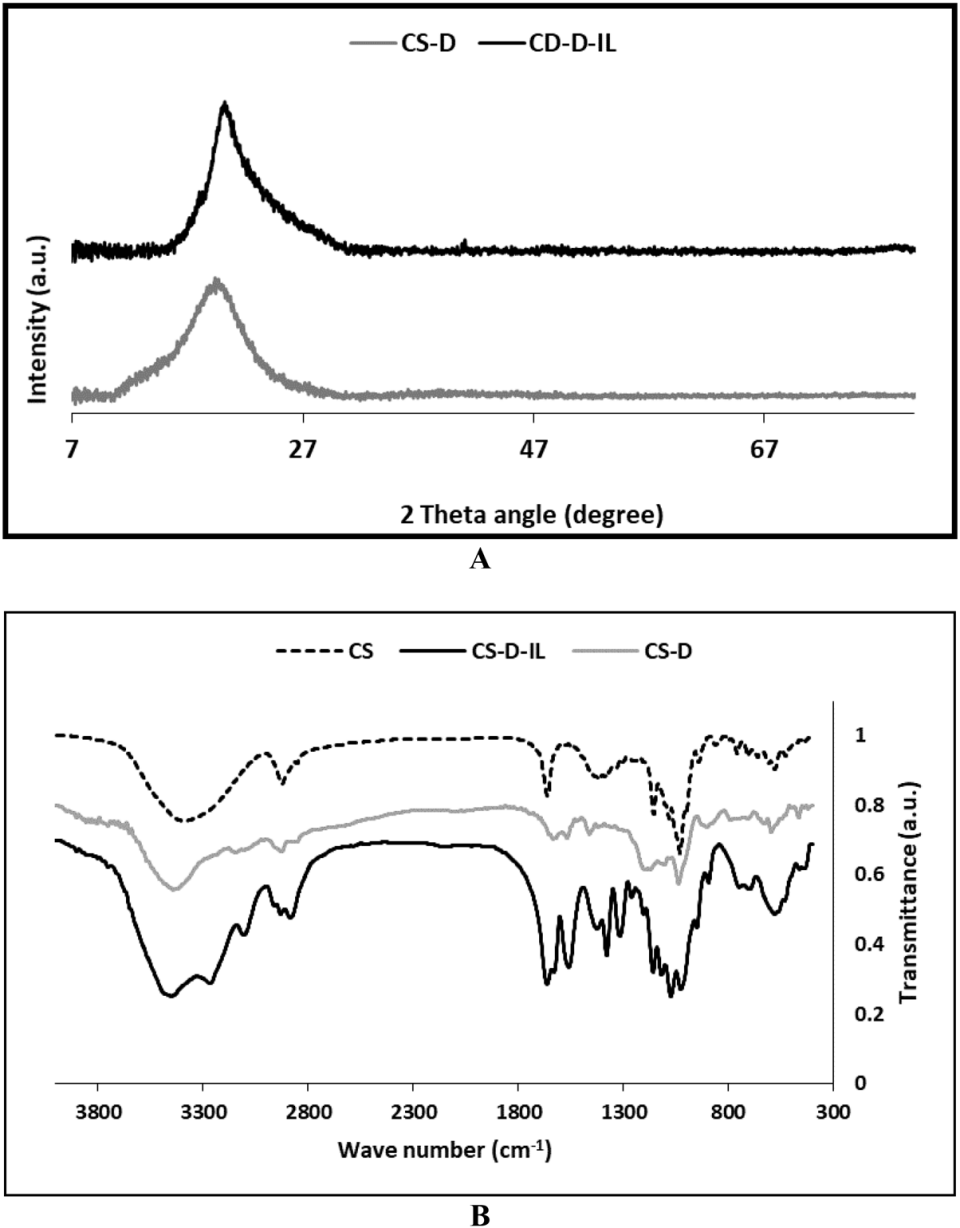
(**A**) XRD patterns of CS-D and CS-D-IL, (**B**) FTIR spectra of CS, CS-D and CS-D-IL.

As CS-D and CS-D-IL possessed no metallic component in their structures, it was expected that these two compounds have amorphous structures. To appraise this issue, XRD analysis of the two samples was performed. The resultant XRD patterns, Fig. 3A, exhibited one peak at 2θ = 21°–25°, approving the amorphous nature of both CS-D and CS-D-IL. This observation is in good agreement with the previous reports^22^.

Using FTIR spectroscopy, CS, CS-D and CS-D-IL were characterized. The characteristic bands of CS are well-known and appeared at 3440 cm^−1^ (–OH functional group), 2917 cm^−1^ (–CH_2_ functionality) and 1656 cm^−1^. Comparing the spectra of CS and CS-D, it can be inferred that the two spectra are very similar and the distinguished bands of CS can be discerned in the spectrum of CS-D, Fig. 3B. This observation is logical, as CS is stable under the working condition for the grafting of D. On the other hand, the characteristic bands of D moiety, i.e. –C=N functionality of TCT groups and –CH_2_ functionality overlapped with the CS bands. The FTIR spectrum of CS-D-IL is also depicted in Fig. 3B. This spectrum is also very similar to that of CS and CS-D. In fact, the main distinguished band in IL that is related to –C=N functionality overlapped with the characteristic bands of TCT. To demonstrate conjugation of D and IL, quantitative analysis can be helpful. In this regard, the investigation of the band appeared at 1656 cm^−1^ (–C=N) in the three spectra confirmed that the intensity of this band increased in the following order: CS-D-IL > CS-D > CS. This result can approve conjugation of TCT in CS-D and IL in the backbone of the catalyst.

To further ascertain the incorporation of D-IL, the TG curves of each intermediate in the course of the synthesis of the catalyst was analyzed by TG analysis. In fact, successful conjugation of each moiety could be verified by TG analysis. The TG curves of CS, CS-D and CS-D-IL are shown in Fig. S1. It was found that, among three TG curves, CS showed the highest thermal stability and contained a weight loss below 150 °C that is due to the loss of water and another one at ~ 300 °C that is attributed to the degradation of CS. Comparing the TG curves of CS-D with that of CS, it can be inferred that the thermal stability of CS-D is lower. This observation is logical as degradation of D that is an organic moiety can decrease the thermal stability. In detail, apart from the observed weight losses of CS, an additional weight loss at 260 °C can be observed in TG curve of CS-D that can be ascribed to the degradation of D. In the TG curve of the catalyst, several weight losses can be observed. The first weight loss is observed due to the loss of water (below 150 °C). After that, a weight loss (41%) is observed at 240–260 °C that can be attributed to the degradation of D-IL. The third weight loss, discerned at ~ 300 °C can be attributed to the degradation of CS.

To evaluate the specific surface area of CS-D-IL, BET analysis was applied. It was found that CS-D-IL exhibited very low specific surface area about 2 m^2^ g^−1^. This value is lower than the specific surface area of untreated CS (4.5 m^2^ g^−1^). The discerned decrement of the specific surface area can approve surface modification of CS.

### Catalyst activity

Considering the environmental concerns, in this research we aimed to devise a novel environmentally benign metal-free heterogeneous catalyst that can promote condensation reaction under mild reaction condition in aqueous media [H_2_O/EtOH (2:1)]. In this regard, promoting Knoevenagel condensation was selected as a model condensation reaction. To prepare the catalyst, CS was selected as a support. The reason for choosing CS was that not only it is a biocompatible and abundant compound, but also the presence of amino functionality imparts catalytic activity to CS to some extent^28^. To start the experiment, the catalytic activity of CS for promoting a model Knoevenagel condensation, reaction of benzaldehyde and malonitrile, was examined in H_2_O/EtOH (2:1) at ambient temperature. The result, Table 1, confirmed that CS showed catalytic activity. However, the reaction time was very long. Then, to improve the catalytic activity and shorten the reaction time, CS was modified by IL. To this purpose, CS was reacted with TCT and 1-methylimidazole successively. The study of the activity of this catalyst, CS-IL, showed that CS-IL exhibited higher catalytic activity compared to CS and could lead to higher yield of the product in shorter reaction time. This issue can be attributed to the instinct catalytic nature of IL^29^. However, the yield of the reaction was still unsatisfactory. To further improve the activity of the catalyst it was decided to improve the loading of IL on CS. In this regard, first a dendritic moiety was grown on CS (see “Experimental” section) to create more sites for the incorporation of IL. TG analyses demonstrated that by incorporation of the dendritic moiety, the IL content increased significantly. Investigation of the catalytic activity of the catalyst (CS-D-IL) also affirmed remarkable improvement in terms of yield of reaction time, Table 1. According to the literature^30^, functional polymers can exert catalytic activity. Hence, to further study the role of the dendritic moiety in the catalysis, another control catalyst, CS-D, was designed and its activity was compared with CS. The result showed higher activity of CS-D compared to CS. This result implied that integration of D in the backbone of CS can improve the activity of the final catalyst. In fact it is possible that the amino functionalities on D promote Knoevenagel condensation through activation of malonitrile.

**Table 1:** Comparison of the catalytic activities of CS-D-IL with some control samples.

Entry	Catalyst	Time (min)	Yield (%)
1	CS-D-IL	90	100
2	CS	360	60
3	CS-IL	150	75
4	CS-D	260	85

Reaction condition: aldehydes (1 mmol), malonitrile (1.2 mmol), catalyst (20 mg) in H_2_O/EtOH (2:1) at 25 °C. b: CS-IL is the catalyst that prepared from reaction of CS with TCT and 1-methylimidazole.

Next, the effects of reaction variables were studied. To study the effect of solvent, the model reaction was performed in water. It was found that only moderate yields of reaction were achieved in water, while use of H_2_O/EtOH (2:1) remarkably increased the reaction yield. It is worth mentioning that further increase of the percentage of ethanol did not affect the yield of the reaction significantly. To elucidate the effect of the catalyst amount and improve the yield of the reaction, various loadings of CS-D-IL were examined for the model reaction. Gratifyingly, increment of the catalyst amount to 20 mg dramatically increased the yield of the product and quantitative yield of the reaction was furnished after 90 min, Table S1. Notably, use of more loading of the catalyst (30 mg) or increase of the reaction temperature to 50 °C slightly accelerated the reaction. Considering the energy and economic issues, the optimum temperature was selected as ambient temperature.

To confirm that the present protocol is not limited to benzaldehyde as a substrate, other aldehydes with electron withdrawing or electron donating functional groups were examined for Knoevenagel condensation. The results can be seen in Table 2. As shown, the present protocol can be generalized to various aromatic aldehydes and the presence of functional groups on the aromatic ring could slightly alter the yield of the desired product. On the other hand, heterocyclic aldehyde could also undergo this reaction to furnish the corresponding product in high yield.

**Table 2:** Knoevenagel condensation reaction between different aldehydes and malonitrile catalyzed by CS-D-IL


Entry	Aldehydes	Yield (%)
1	Benzaldehyde	100
2	4-NO_2_-benzaldehyde	100
3	4-Cl-benzaldehyde	100
4	3-NO_2_-benzaldehyde	95
5	4-Me-benzaldehyde	100
6	2-NO_2_-benzaldehyde	95
7	4-MeO-benzaldehyde	98
8	2-MeO-benzaldehyde	95
9	Furfural	90

Reaction condition: aldehydes (1 mmol), malonitrile (1.2 mmol), Catalyst (20 mg) in H_2_O/EtOH (2:1) at 25 °C.

**Figure 4:**
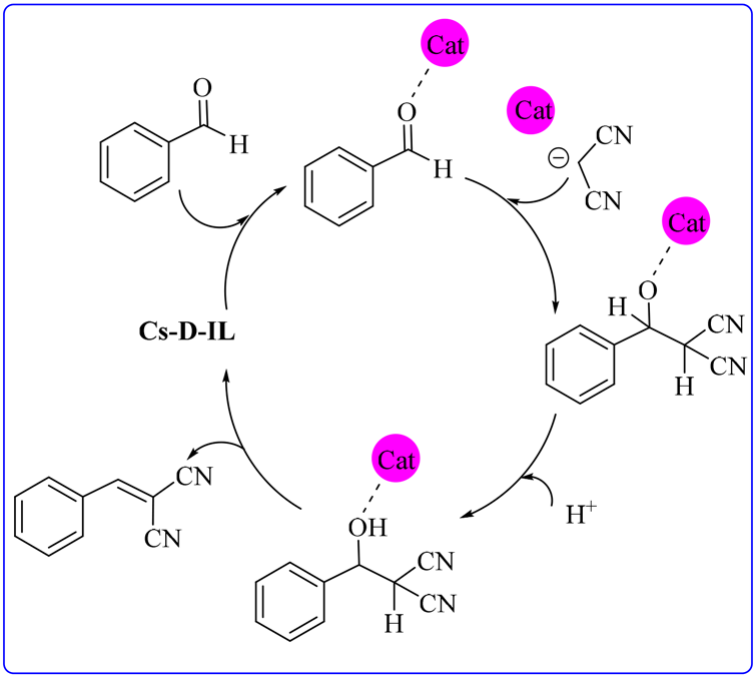
The plausible mechanism for Knoevenagel condensation.

Regarding the reaction mechanism, it can be suggested that CS-D-IL can activate the carbonyl group in the aldehyde^31^. Meanwhile, the counter ion of IL in the backbone of the catalyst can activate malononitrile. The reaction of the two activated starting materials will form an intermediate that generate the desired product and free CS-D-IL, Fig. 4.

In the next part of the study of the catalytic activity, the performance of CS-D-IL for Knoevenagel condensation of benzaldehyde and malonitrile was compared with some other methodologies, Table S2. Considering the tabulated data, it can be inferred that both metal free and metallic catalysts have been reported for this condensation reaction. Considering the high cost of precious metals and environmental concerns, use of available, low cost and bio-based metal free catalyst is of great attraction. On the other hand, use of heterogeneous catalyst is more favorable than homogeneous ones in terms of recycling and ease of work-up procedures. Synthesis of some other catalysts such as metal organic frameworks requires precise protocols and possibly costly reagents. To sum up, although exact comparison among various methodologies cannot be fulfilled due to the different reaction condition, it can be inferred that CS-D-IL is among highly active catalysts that can promote the Knoevenagel condensation under very mild reaction condition efficiently.

Motivated by high catalytic activity of CS-D-IL for Knoevenagel condensation, it was appraised whether other chemical transformations can be promoted by CS-D-IL. In this regard, one-pot two component reactions of aldehydes with dimedone for the synthesis of xanthenes were examined under CS-D-IL catalysis. First, the reaction condition was optimized. It was found that by using 30 mg CS-D-IL in H_2_O/EtOH (2:1) as solvent the reaction proceeded at 50 ºC to give the product in high yield after 3 h. Notably, investigation of the generality of this methodology established that various aldehydes with different electron density can tolerate the reaction to furnish the corresponding product in high yield, Table 3. Even heterocyclic aldehyde such as furfural could also undergo this reaction. However, the yield of the reaction was slightly lower than non-aromatic counterparts.

**Table 3:** Synthesis of various xanthenes under CS-D-IL catalysis .


Entry	Substrate	Yield (%)^a^
1	Benzaldehyde	95
2	4-NO_2_-benzaldehyde	95
3	2-NO_2_-benzaldehyde	90
4	4-Me-benzaldehyde	93
5	4-MeO-benzaldehyde	92
6	2-MeO-benzaldehyde	90
7	4-Cl-benzaldehyde	95
8	Furfural	80

^a^Isolated yield.

**Figure 5:**
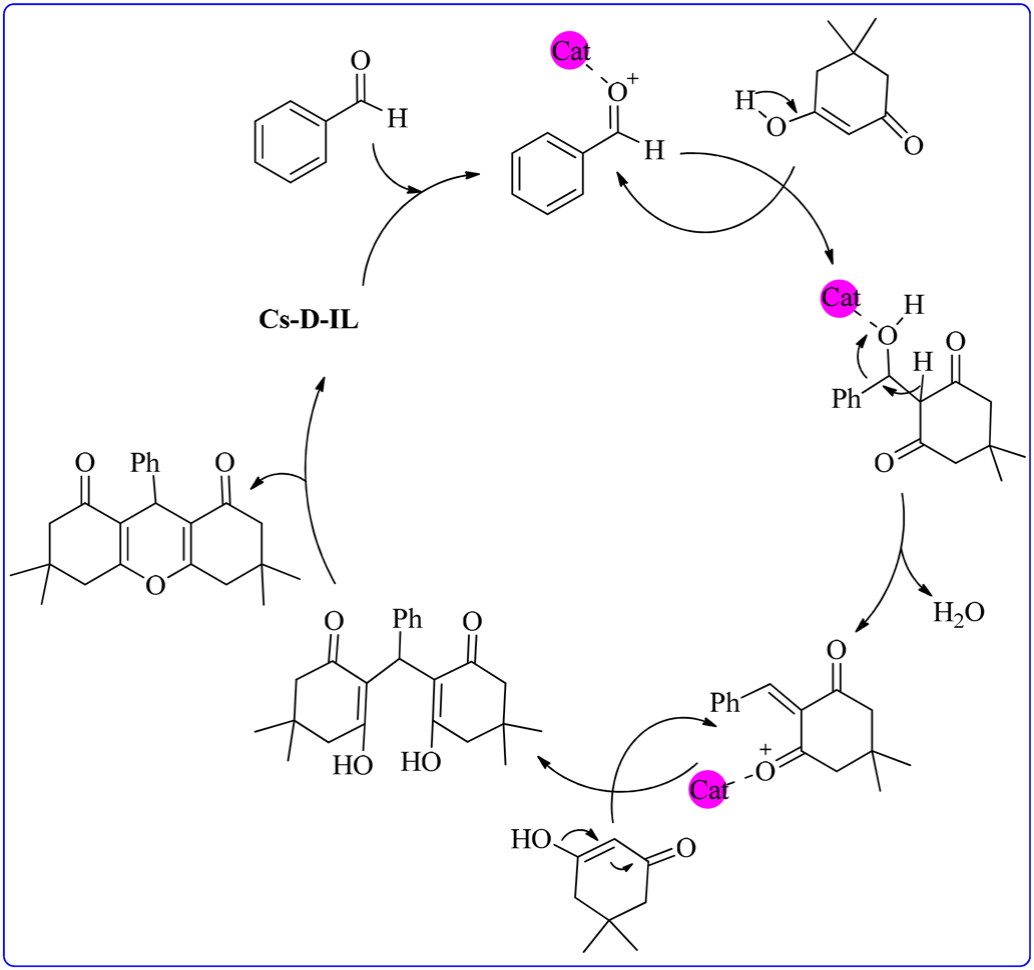
The plausible mechanism for xanthene synthesis.

The possible mechanism for xanthene synthesis is depicted in Fig. 5. Similar to the Knoevenagel condensation, the reaction proceed through activation of carbonyl group of the aldehyde. Subsequently, the enole form of dimedone tolerated reaction with the activated aldehyde to produce an intermediate. The latter then dehydrated and reacted with the second dimedone. Finally, xanthene will be achieved via dehydration and cyclization^32^.

### Catalyst recyclability

**Figure 6:**
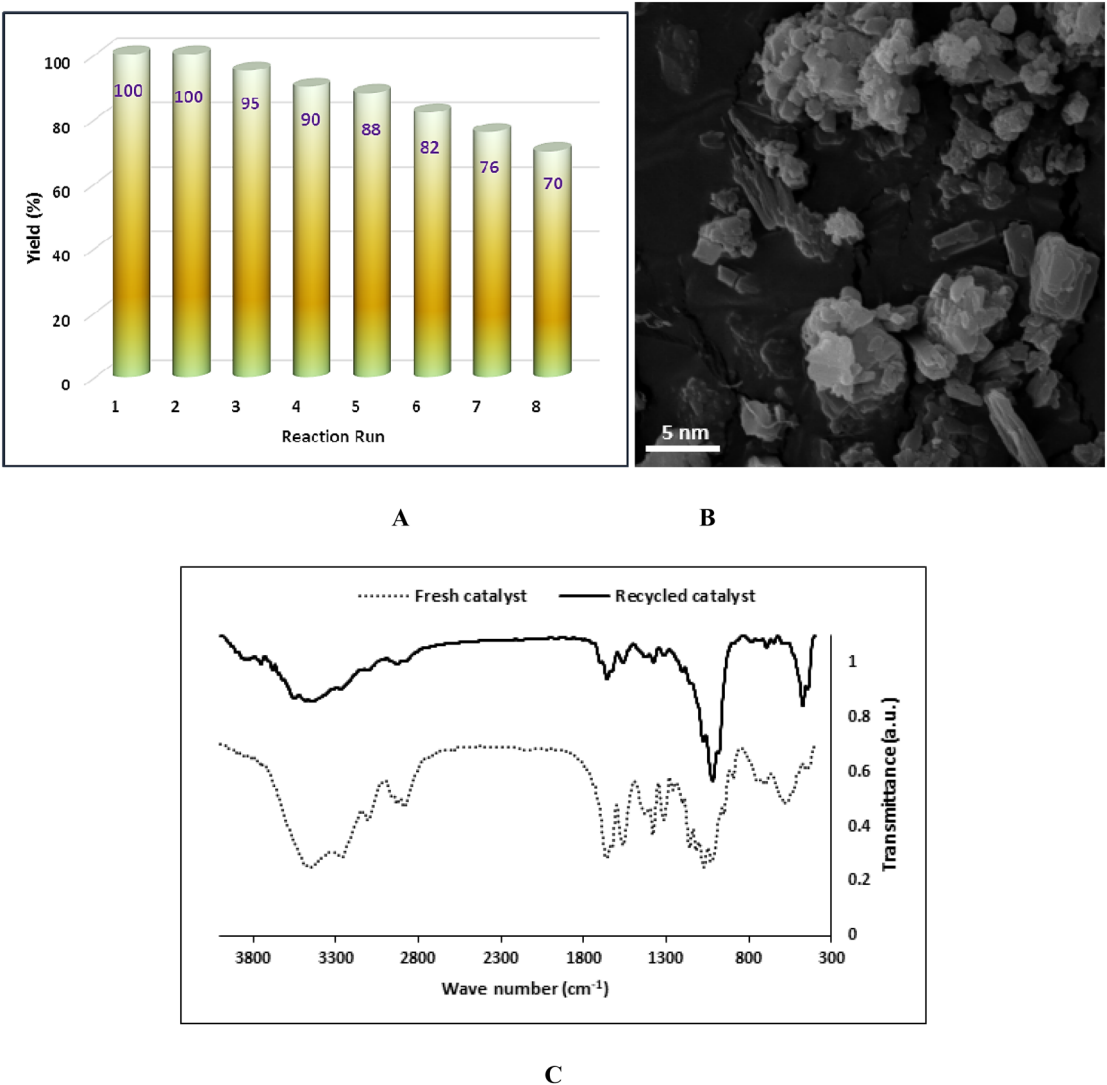
(**A**) The recyclability of CS-D-IL, (**B**) SEM image of the recycled CS-D-IL and (**C**) the comparison of FTIR spectra of fresh and recycled catalysts.

In the final part of this research, one of the most important feature of a heterogeneous catalyst, i.e. recyclability was appraised. In fact the success of the present bio-based and metal free catalyst lies in efficient recyclability. Again, the reaction of benzaldehyde and malonitrile was selected as a model catalyst and the recyclability of the catalyst was examined for this reaction for eight runs. Gratifyingly, the results ascertained that CS-D-IL maintained its activity for the second run of the condensation reaction and after that, slight catalytic loss was discerned up to fifth runs. Then, the loss of the activity of the catalyst accelerated and 30% loss of the catalytic activity was observed after eighth run, Fig. 6A.

To elucidate how recycling can affect the morphology of CS-D-IL, the recycled CS-D-IL after eight runs was characterized by SEM. As illustrated in Fig. 6B, the morphology of the recycled CS-D-IL is distinguished from the fresh catalyst. The observed morphological change can be attributed to the several washing steps in the course of recycling. In Fig. 6C, the comparison of the FTIR spectra of fresh and recycled CS-D-IL is presented. This comparison signifies that all of the characteristic bands of fresh CS-D-IL can be discerned in the spectrum of the recycled catalyst. This issue is indicative of the structural stability of the catalyst upon reusing.

## Experimental

### Materials

The reagents used for the fabrication of CS-D-IL and examining its activity for Knoevenagel condensation and synthesis of xanthene derivatives included, CS (Mw = 50,000–190,000, with deacetylation degree ≥ 75%, viscosity 20 cP for 1 wt%, in 1% acetic acid), 2,4,6-trichloro-1,3,5-triazine (TCT), ethylenediamine (EDA), 1-methylimidazole, tetrahydrofuran (THF), acetic acid, aromatic aldehyde, malonitrile, dimedone, EtOH, distilled water. All of the mentioned reagents were purchased from Sigma-Aldrich and applied as received. The details of the instruments used for verification of the catalyst are presented in SI.

### Fabrication of the catalyst

#### Synthesis of TCT functionalized chitosan: synthesis of CS-TCT

To prepare CS-TCT, a solution of CS (3 g) in 100 mL acetic acid (2%) was agitated at room temperature for 5 h. Then, a solution of TCT (2 g) in THF (20 mL) was added into the CS solution and the obtained mixture was stirred at 25 ºC overnight. Upon completion of the reaction, the solvent was evaporated and the solid product was washed with THF for three times to remove unreacted TCT. CS-TCT was then dried at ambient temperature overnight and applied for the next step of synthesis of the catalyst.

#### Growing dendron on the CS-TCT: synthesis of CS-D

To grow dendron, EDA (2 mL) was added to the mixture of the as-prepared CS-TCT (2 g) in THF (60 mL). To assure successful substitution reaction of EDA with CS-TCT, the mixture was kept under reflux condition for 12 h. At the end of the reaction, CS-TCT-EDA was filtered by centrifuging at 4000 rpm, washed with THF repeatedly and dried at 60 ºC for 8 h. In the second step, the as-prepared CS-TCT-EDA (1.5 g) and TCT (1.5 g) were placed in THF (50 mL) and agitated at 25 ºC overnight. Afterwards, the product was filtered and washed with THF.

#### Grafting imidazolium salt on CS-D: synthesis of CS-D-IL

CS-D (1 g) was suspended in THF (60 mL) and stirred vigorously. Then, 1-methylimidazole (1 g) was added to the aforementioned suspension and the obtained mixture was heated up to 70 °C and stirred for 24 h. At the end of the reaction, the precipitate was filtered off, washed with THF several times and finally dried under vacuum at 60 °C. In Fig. 1, the schematic representation of the synthetic procedure of CS-D-IL is illustrated. The procedures used for Knoevenagel condensation and synthesis of xanthenes are elaborated in SI.

## Conclusion

To address one of the main challenges of chitosan supported ILs, i.e. low loading of ILs, a solution is reported by introduction of dendritic moiety. In fact, a metal free catalytic composite composed of CS, IL and dendritic moiety has been reported through growth of dendron on CS via successive reactions of TCT and EDA and decoration of terminal groups with IL. It was postulated that the presence of dendritic moiety can allow conjugation of more ILs on the backbone of the catalyst. To confirm this assumption, the catalytic activity of the catalyst for Knoevenagel condensation in aqueous media under mild reaction condition was assessed and compared with dendritic free catalyst. The results confirmed the assumption and established lower IL loading of dendritic free catalyst and its lower catalytic activity. In fact, the dendritic moiety could furnish an opportunity to increase the loading of IL and consequently improved the catalytic activity of the final catalyst. The results indicated high activity of the catalyst, superior to some previously reported catalysts. Moreover, the utility of the present catalyst for the synthesis of xanthene derivatives was also confirmed. Notably, the present catalyst could be recycled and reused for several runs. These results affirmed that CS-D-IL is a potential metal-free catalyst that benefits from high loading of IL and can be considered as a versatile catalyst for promoting chemical transformation in aqueous media under mild reaction condition.

## Supplementary information

Supplementary Information.
