# Supplementary material for: Fabrication of a metal free catalyst for chemical reactions through decoration of chitosan with ionic liquid terminated dendritic moiety
Source: Sci Rep. 2020 Nov 12;10:19666. doi: 10.1038/s41598-020-76795-8 (PMC7661698; doi:10.1038/s41598-020-76795-8)
Supplement: Supplementary file 1 — Supplementary Information. [file 41598_2020_76795_MOESM1_ESM.docx]

**Supporting information**

**Fabrication of a metal free catalyst for chemical reactions through decoration of chitosan with ionic liquid terminated dendritic moiety**

*Samahe Sadjadi^*1^, Fatemeh Koohestani^1^, Majid M Heravi^2*^*


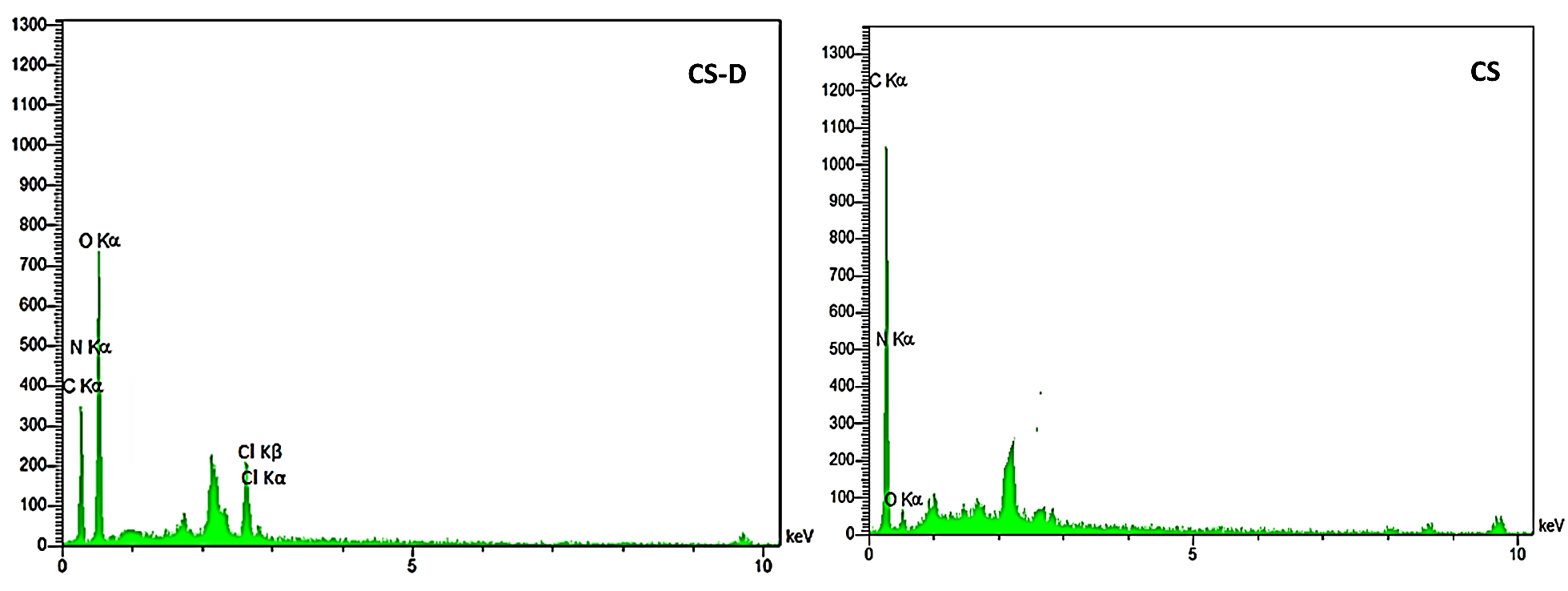


**Figure S1**, EDS analysis of CS and CS-D.
